# Investigating Gaze Behaviour of Children Diagnosed with Autism Spectrum Disorders in a Classroom Setting

**DOI:** 10.1007/s10803-021-04906-z

**Published:** 2021-02-15

**Authors:** Aideen McParland, Stephen Gallagher, Mickey Keenan

**Affiliations:** 1 Ulster University, Coleraine, Northern Ireland UK; 2 Present Address: School of Psychology, Queen’s University Belfast, David Keir Building, Belfast, UK; 3 School of Psychology, Ulster University, Cromore Road, Coleraine, UK

**Keywords:** Autism, Eye-tracking, Gaze behaviour, Behaviour change, Social skills, Applied behaviour analysis

## Abstract

A defining feature of ASD is atypical gaze behaviour, however, eye-tracking studies in ‘real-world’ settings are limited, and the possibility of improving gaze behaviour for ASD children is largely unexplored. This study investigated gaze behaviour of ASD and typically developing (TD) children in their classroom setting. Eye-tracking technology was used to develop and pilot an operant training tool to positively reinforce typical gaze behaviour towards faces. Visual and statistical analyses of eye-tracking data revealed different gaze behaviour patterns during live interactions for ASD and TD children depending on the interaction type. All children responded to operant training with longer looking times observed on face stimuli post training. The promising application of operant gaze training in ecologically valid settings is discussed.

## Introduction

Atypical gaze behaviour when viewing social stimuli is a key characteristic of autism spectrum disorders (ASD) (Chawarska et al. 2013; Noris et al. 2012; Rice et al. 2012). Gaze—where one looks, how long, and when—plays an essential part in human social behaviour and is therefore an important consideration to better understand the challenges that individuals with ASD typically face when initiating and regulating social interactions (Hessels 2020). Atypical eye-gaze among individuals with ASD has been linked to challenges with recognising face identity (Parish-Morris et al. 2013), emotional expressions (Bal et al. 2010) and often interferes with assigning social relevance to gazed-at objects (Riby et al. 2013; Vivanti et al. 2011). Knowing the short and long-term impacts of atypical gaze behaviour for individuals with ASD, only strengthens the importance to learn more about this unique behaviour in daily environments that individuals with ASD find themselves in. It also allows the possibility of helping individuals with ASD at a young age to overcome some of these challenges using eye-gaze training as a starting point (Chita-Tegmark 2016) which will allow potential longer-term benefits to be monitored during the course of their development.

Previous eye-tracking research has been characteristically thwarted in its scope of presenting individuals with ASD either static or dynamic representations of social stimuli on a computer screen when assessing gaze behaviour (for review see Guillon et al. 2014; for meta-analysis see Chita-Tegmark 2016). Based on this experimental paradigm, researchers have been relatively split in their findings, often reporting a more typical pattern of gaze behaviour when viewing static social stimuli (Anderson et al. 2006; Chawarska and Shic 2009; Dalton et al. 2005; Elsabbagh et al. 2013; Freeth et al. 2010; Key and Stone 2012; Riby and Hancock 2009; Van der Geest et al. 2002; Wilson et al. 2010) and an atypical gaze behaviour pattern when viewing dynamic social stimuli that involves motion (Hosozawa et al. 2012; Klin et al. 2002; Rice et al. 2012; Shic et al. 2011; Speer et al. 2007).

However, neither of these eye-tracking designs allow ecologically valid conclusions to be drawn in their absence of naturalistic face to face interactions; a significant and necessary consideration given that human interactions and environments heavily dictate our gaze behaviour (Chita-Tegmark 2016; Guillon et al. 2014). Indeed, over the last number of years, there has been a general consensus amongst researchers that the traditional laboratory studies that have focused on social attention or social gaze have misrepresented how gaze may operate in ‘real-world’ situations (e.g. Birmingham et al. 2008; Cole et al. 2016; Smilek et al. 2006; Hayward et al. 2017). This has led to increasing calls to action to include realistic, ecologically valid stimuli in eye-tracking research (recommended by Chita-Tegmark 2016; Hessels 2020).

## Live Social Interactions in Eye-Tracking Research

The use of live face to face interactions when assessing gaze behaviour of individuals with ASD is a relatively new approach in eye-tracking research, made possible with advances in remote eye-tracking technology over the last 6 years (Falck-Ytter 2015). It has been hailed as an innovative way to overcome many of the general biases and task-dependent inaccuracies consummate with static or dynamic stimuli, since it captures the main building blocks integral to authentic social interactions; talking, moving, eye-movement, eye-contact, distractions etc. (Hessels 2020). A study by Noris et al. (2011) was the first to assess the gaze behaviour of children with ASD (3–9 years old) during a dyadic interaction in a naturalistic setting. Researchers used a head-mounted ‘WearCam’ worn by each participant to record the field of view as seen by the child, and to simultaneously record the gaze direction of the child. The dyadic interaction required participants to blow soap bubbles, play with a mechanical toy, play with a toy car, and play with a small ball presented to them by the experimenter (Noris et al. 2011). Researchers found that children with ASD looked downwards more often and explored their lateral field of view more extensively than typically developing (TD) controls during the interaction (Noris et al. 2011). This was explained in relation to the phenomenon of downcast gaze in autism (Bogdashina et al. 2005) which is considered to be a response to sensory overload in the environment and hypersensitivity to real life visual stimuli (Noris et al. 2011).

Supporting empirical evidence for this study was conducted by Falck-Ytter (2015). This study aimed to quantify the amount of time spent looking at another person’s face during face-to-face communication in children with ASD (*M*_age_ = 6 years) and TD controls (*M*_age_ = 6 years). Using a head mounted eye-tracking camera (Tobi TX300; 60 Hz), the researcher found that in the context of high ecological relevance—listening to an adult telling a story—children with ASD showed a markedly reduced tendency to look at the adult’s face (Falck-Ytter 2015). This finding was speculated to relate to the social/communicative intent of this type of interaction; the adult looked often at children and asked them questions. According to Falck-Ytter (2015), this resulted in an inability of children with ASD to regulate one’s own looking; children with ASD preferred to look away at distinct times when the adult would ask them questions about the story (Falck-Ytter 2015). The impact of communicative intent on gaze behaviour when engaged in live interactions with individuals with ASD continues to gather empirical support. A recent study by Vabalas and Freeth (2016) reinforced the significance of communicative intent with their findings that ASD students were more likely to avert gaze and demonstrate less frequent saccades when an actor was talking to them directly. Given the impact that communicative intent has increasingly been found to have when measuring gaze behaviour during live interactions, it played a key part in the design of the current study.

Previous eye-tracking studies detailed here use live face to face interactions with children (and adults) with ASD, uncovering novel findings of gaze behaviour amongst this population when effectively re-creating play time situations or using actors to simulate conversations. However, can this research conducted in a highly controlled and staged lab environment be described as ‘real-world’ research? According to a recommendation by Weisz (2000) atypical behaviours “….*should be tested in the context in which they are used, from the beginning, without the many controlled settings used in ASD research”* (Weisz 2000, p. 837). This is something the current study wished to address by conducting an eye-tracking experiment in a child’s natural, daily environment—their school classroom.

## Improving Gaze Behaviour Using ABA

In addition to previous eye-tracking research lacking in ‘real-world’ experimental designs, we also recognised the potential for *improving* gaze behaviour during live social interactions for children with ASD. It is our contention this should begin to bridge the research to practice gap by developing a training tool that would help children with ASD attend to socially relevant stimuli in their natural environments and improve communication skills. The science of Applied Behaviour Analysis (ABA) has consistently offered the most effective basis for the treatment of ASD (for review see Medavarapu et al. 2019). A study conducted by Isaksen and Holth (2009) highlighted the fruitful benefits of this endeavor. These researchers targeted certain joint attention skills (turn-taking, responding to joint attention bids, initiating joint attention bids) amongst four children diagnosed with ASD. They developed certain training procedures to establish adult social responses (smiling and nodding) as conditioned reinforcers. Contingent upon the child looking in the same direction as an adult, the teacher nodded and smiled, and the child was allowed to play with objects on the table (Isaksen and Holth 2009). Researchers found that all four children made significant progress engaging in joint attention and initiating joint attention as a result of positively reinforcing these social cues.

Since gaze behaviour has long been considered an operant behaviour capable of changing in response to reinforcing contingencies (Dube et al. 2010; Schroeder and Holland 1968; Schroeder 1970; Steingrimsdottir and Arntzen 2016), this formed the scientific grounding on which an operant training tool could be developed in the current study. This operant approach to behavioural interventions with ASD populations has also been advocated by Taylor and Hoch (2008) who commented that “*future studies may want to determine what tangible rewards can be paired with social interaction to create a conditioned reinforcer and therefore behaviour change for children with Autism.”* (Taylor and Hoch 2008, p. 380).

## The Current Study

The overall aims of the current study were two-fold. Firstly, to promote ecological validity in eye-tracking research with ASD children, this study aimed to measure gaze behaviour amongst children with ASD in their classroom, a familiar and natural environment. Part of this aim included investigating further the issue of communicative intent (Falck-Ytter 2015; Vabalas and Freeth 2016) by involving children with ASD in a dyadic interaction (high in communicative intent) and a triadic interaction (low in communicative intent). The second aim of this study was to pilot the use of eye-tracking technology to develop an operant training tool rooted in the scientific principles of ABA and develop social reinforcing contingencies akin to research by Isaksen and Holth (2009). We aimed to map the traditional three term contingency of operant conditioning-Antecedent-Behaviour-Contingency (ABC) (Skinner 1969) onto a training procedure ran on eye-tracking equipment. The eye-tracking equipment was configured to present social stimuli on a computer screen (Antecedent) and once a participant looked at an area of interest—the face (Behaviour), this triggered a reward screen reinforcing this target behaviour by winning points on a reward chart (Consequence). The face was chosen to act as the area of interest because of the evidence base that suggests gaze to faces significantly supports effective understanding of live, social interactions (Hessels 2020). Linked to this second aim of developing an operant training tool, a single-subject research design was used so that each participants’ unique gaze behaviour could be measured across experimental phases without being lost in comparative between- group data analyses—a common pitfall of eye-tracking research with ASD populations (Kazdin 2019).

Two research questions transpired; (1) does an atypical gaze behaviour pattern previously found amongst children with ASD during live interactions, occur in a ‘real-world’ classroom setting? and (2) can gaze behaviour be improved in this ‘real-world’ setting using an operant training tool?

## Methods

### Participants

Twenty primary school children (*n* = *20)* aged between five and eleven years old (*M*_age_ = 7.9 years old; *SD* = 1.62) were recruited from a mainstream primary school located in Northern Ireland. Ten children (7 males, 3 females) had received a clinical diagnosis of ASD and the remaining ten children (6 males, 4 females) were typically developing (see Table 1). Six of these twenty primary school children (3 ASD and 3 TD) were randomly assigned to act as control participants who did not complete operant training during the experimental session. Parental consent on behalf of each child was provided prior to the experiment commencing, and verbal assent was given by each participant on the day of the experiment. Ethical approval was obtained via the relevant university ethics committee, and testing was conducted in line with the British Psychological Society guidelines.

**Table 1:** Participant characteristics

	Demographic information							
Pseudonym	Age	ASD/TD	Gender	SRS *T*-Score^a^	Pseudonym	Age	ASD/TD	Gender
Brian	9 years	ASD	Male	83(S)	Olivia	7 years	TD	Female
Rosie	5 years	ASD	Female	62(M)	Catherine	11 years	TD	Female
Connor	7 years	ASD	Male	78(S)	Neil	10 years	TD	Male
Michael	6 years	ASD	Male	66(Mo)	Ross	8 years	TD	Male
Ben	6 years	ASD	Male	64(M)	Stephen	8 years	TD	Male
Clare	8 years	ASD	Female	85(S)	Susan	9 years	TD	Female
Joseph	10 years	ASD	Male	60(M)	Julie	6 years	TD	Female
Jack^b^	8 years	ASD	Male	79(S)	Ryan^b^	7 years	TD	Male
Charlie^b^	7 years	ASD	Male	84(S)	John^b^	10 years	TD	Male
Emma^b^	9 years	ASD	Female	74(Mo)	Luke^b^	7 years	TD	Male

^a^SRS-2 ASD symptom severity range in parenthesis; *M* mild, *Mo* moderate, *S* severe

^b^Demarks control participant who did not complete operant training

### Apparatus and Materials

SMI (Senso Motoric Instruments, Tetlow, Germany) remote eye-tracking glasses were used for data recording during live interactions. Two small cameras on the rim of the glasses captured the eye movements of the wearer, and the recorded gaze fixations were mapped onto the scene camera video on the researchers SMI Eye-Tracking Glasses laptop (SMI-ETG laptop) via the iView X™ (Inition, London, UK) dark pupil tracking system. The range of eye-tracking with these glasses was 80° horizontal, 60° vertical and up to 0.1° spatial resolution. A one-point calibration procedure was used. Real-time accuracy of calibration was assessed by observing the location of the participants’ gaze fixation (represented as a circular cursor on SMI-ETG laptop screen) and mapping this onto the visual field view recording. A SMI desktop-based eye-tracking device was also used to deliver operant training. This SMI eye-tracking equipment had a sampling rate of 500 Hz. A five-point calibration procedure was used—this required a participant to look at five consecutive points on the computer screen. Data recorded when using the SMI eye-tracking glasses were stored on the SMI-ETG laptop, and data recorded when using the desktop-based eye-tracker, were stored on the SMI laptop. All analyses of recorded eye-tracking data were conducted using SMI BeGaze™ software.

Materials used in this study included a reward chart and flash cards. The reward chart and corresponding flash cards acted as positive reinforcement to each participants’ gaze behaviour when they met the criterion of looking at an image of a face presented during a dynamic video clip for one second. A reward chart was used as reinforcement due to participants’ familiarity of using this as positive reinforcement in their school day and its common use in ABA practice. A timer was also used to ensure equal durations of live interactions during baseline and re-test phases.

### Stimuli

**Fig. 1:**
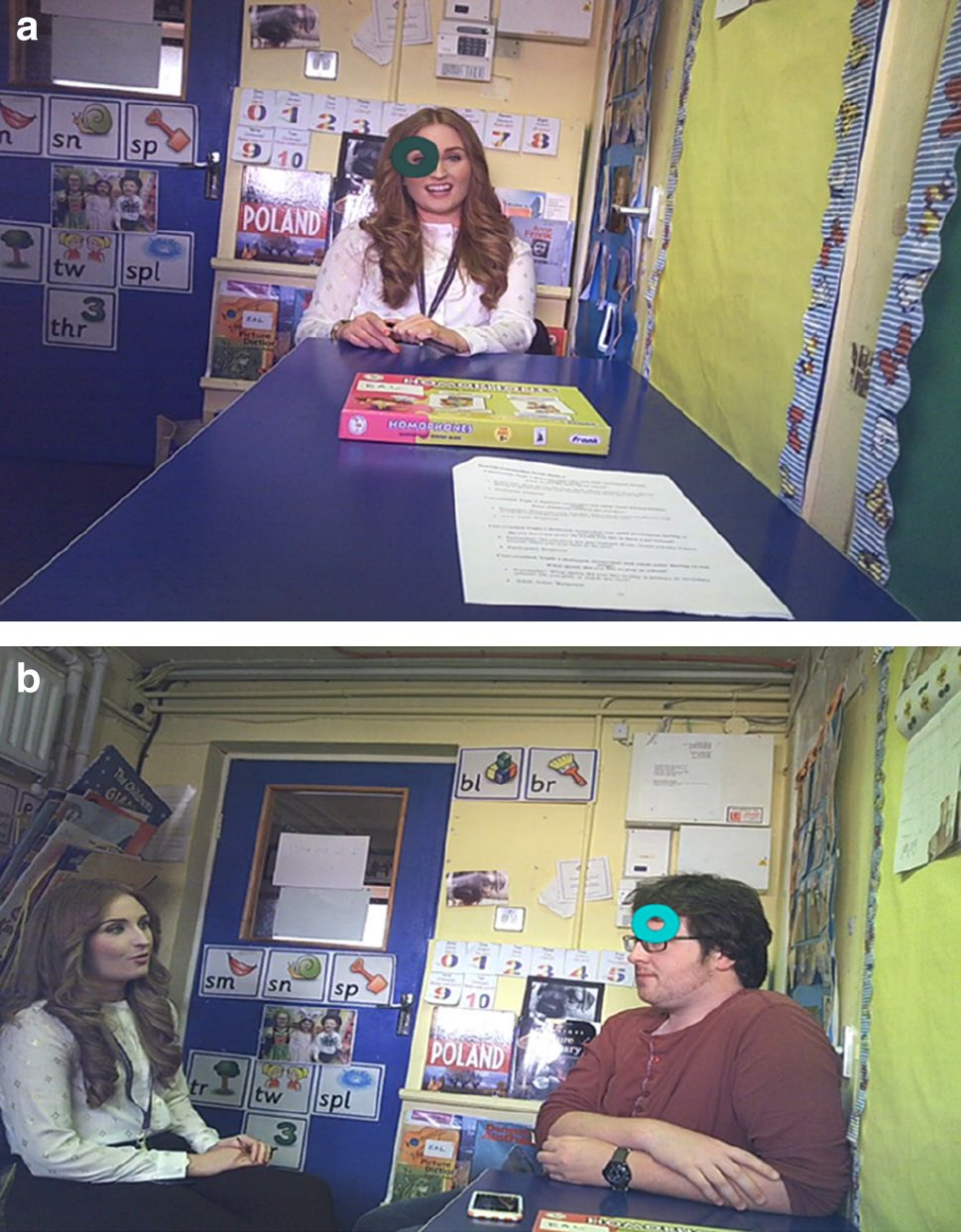
**a** Participants’ perspective during a live dyadic social interaction. **b** Participants’ perspective during a live triadic social interaction

Eye-movements were recorded for each participant (using SMI eye-tracking glasses) during a live set of social interactions which took place in a learning support classroom in their primary school. Social stimuli during these live interactions consisted of the researcher engaging in a conversation with the child (dyadic interaction) and the researcher and research assistant engaging in a conversation with the participant watching/contributing to the conversation (triadic interaction). Figure 1 shows an example of each type of social interaction which was conducted in this ‘real-world’ school setting. A pre-determined conversation dialogue was agreed between the researcher and research assistant to ensure content consistency. These conversation topics began with leading questions; *‘what is your favourite thing about school?’ (dyadic interaction), ‘what sports did you enjoy most when you were younger?’ (triadic interaction), ‘what are your favourite animals?’ (dyadic interaction), and ‘what was your favourite cartoons to watch as a child?’ (triadic interaction).*

**Fig. 2:**
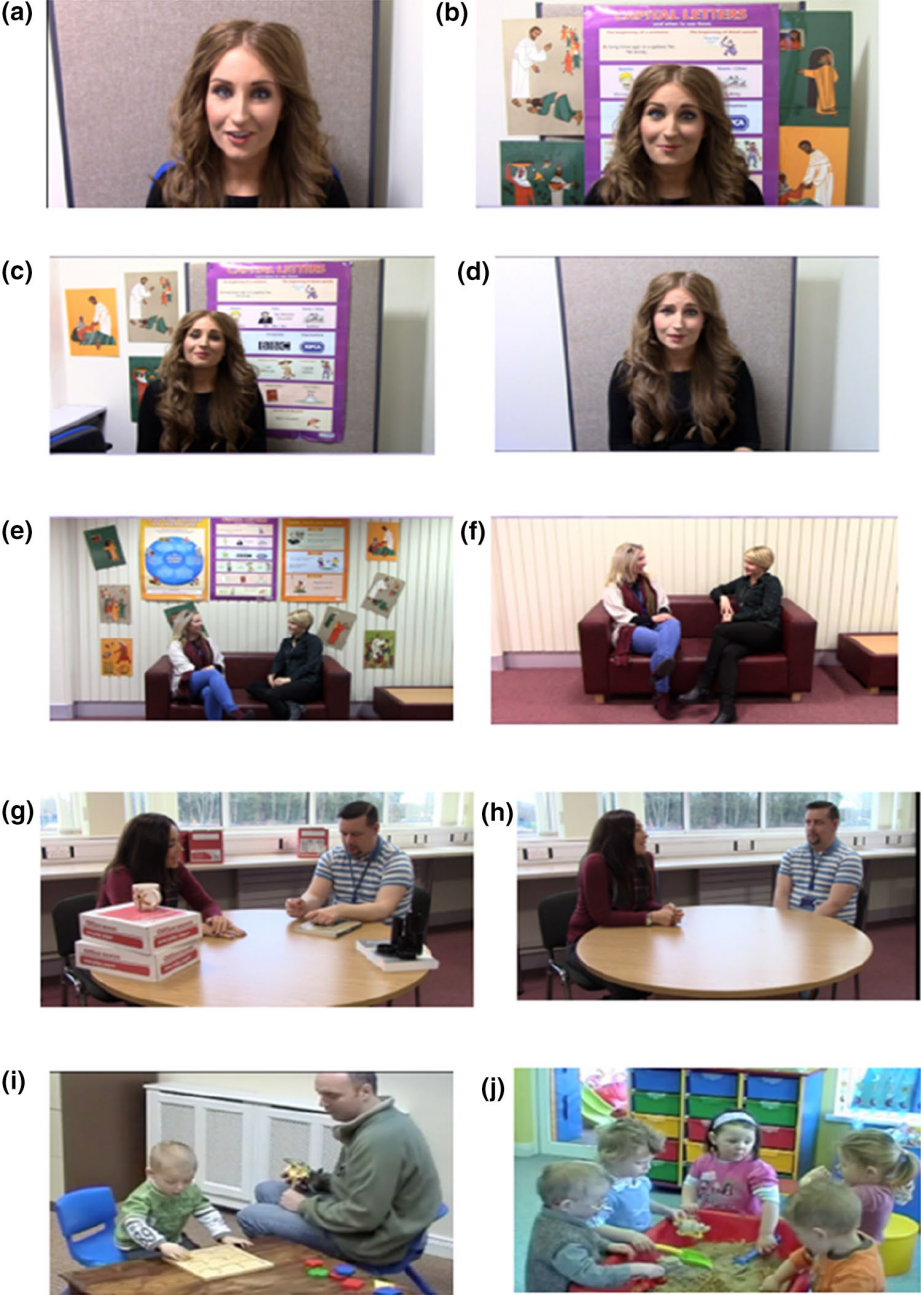
Stills of dynamic stimuli; **a** single actor head shot, **b** single actor head shot (distractors), **c** single actor waist up (distractors), **d** single actor waist up, **e** female–female interaction (distractors), **f** female–female interaction, **g** female–male interaction (distractors), **h** female–male interaction, **i** adult–child interaction, **j** children playing

In addition to these live social stimuli, dynamic social stimuli presented during the operant training phase consisted of 10 short video clips depicted in the following categories presented in Fig. 2; (1) Female–Female interaction, (2) Female–Male interaction, (3) Single actor (head shot), (4) Single actor (waist up), (5) Adult–Child interaction, (6) Children playing.

## Measures

A measure of social functioning for each participant who had received a clinical diagnosis of ASD was obtained for this study using the Social Responsiveness Scale (SRS 2nd Edition) (Constantino and Gruber 2005). This was a 65-item rating scale that measured the severity of autism spectrum symptoms as they occur in a natural social setting using a ‘1’ (not true) to ‘4’ (almost always true) Likert rating scale. The SRS has a high reported internal consistency with a Cronbach’s alpha coefficient of 0.95 (Constantino and Gruber 2005). Cronbach’s alpha in the current ASD sample was 0.89. The SRS-2 provided a clear picture of a child’s social impairments across five subscales: social awareness, social cognition, social communication, social motivation, and restricted interests and repetitive behaviours (Constantino and Gruber 2005). The questions focused on the child’s behaviour during the past 6 months with higher scores indicating greater impairments. Since participants were under the age of 18, the SRS-2-School-Age form was used and each teacher who had a child diagnosed with ASD in his/her class completed the assessment. Raw scores were converted to *T*-Scores and interpreted as: ≤ 59* T*, within normal limits; 60–65 *T*, mild; 66–75 *T*, moderate; ≥ 76* T* severe social impairments. *T*-scores were used in the analysis for the current study.

## Design

A single- subject A-B experimental design with a training phase prior to the B phase was used. This design was used as opposed to other single-subject research designs due to the time constraints of a single allocated 30-min session with each participant during their school day. The A-B design allowed an accurate, rapid, measurement of gaze behaviour during the three experimental stages. The current study reflected recent standards outlined by What Works Clearinghouse (2020) on conducting single- subject studies: (1) participants comprised of a cluster from the same primary school, with each participant being the unit of measurement for intervention and data analysis (2) the outcome variables of duration, frequency and latency of gaze behaviour were measured prior, during, and after the intervention/training phase (3) the outcome variables were measured repeatedly in multiple conditions/trials throughout each experimental phase (4) no overlaps existed between the training phase—each participant completed the experimental phases successively (5) at least three data points were used in this study (baseline, training, re-test) to demonstrate experimental effect (6) participants were randomly assigned as control participants to ensure internal validity and prevent confounding factors being associated with the effectiveness of the operant training tool.

## Procedure

### Baseline Phase

During the baseline phase, each participant firstly engaged in a *dyadic interaction* with the researcher. Wearing the eye-tracking glasses, the participant was asked by the researcher, ‘*what is your favourite thing about school?’.* The researcher and participant spoke for a duration of 2 min 30 s. This was timed using a timer by the research assistant. Next, the researcher and research assistant sat facing each other while the participant remained seated on the opposite end of a desk *in a triadic interaction*. The researcher and research assistant began to talk about ‘*what were your favourite cartoons as a child?’.* The participant was free to contribute to the conversation if he/she wished. Participants completed this baseline phase within five minutes.

### Training Phase

Prior to testing in the primary school, researchers used the eye-tracker’s dwell time trigger mechanism to create a behaviour contingency during the training phase. This was created by drawing a box around dynamic face stimuli that would be presented to participants on the eye-tracking monitor. This box was configured so that each time a participant fixated within this box for one second, it would trigger the next screen to appear (a centre cross screen). Participants could not see this box during the experimental phase and were not aware of any trigger box.

**Fig. 3:**
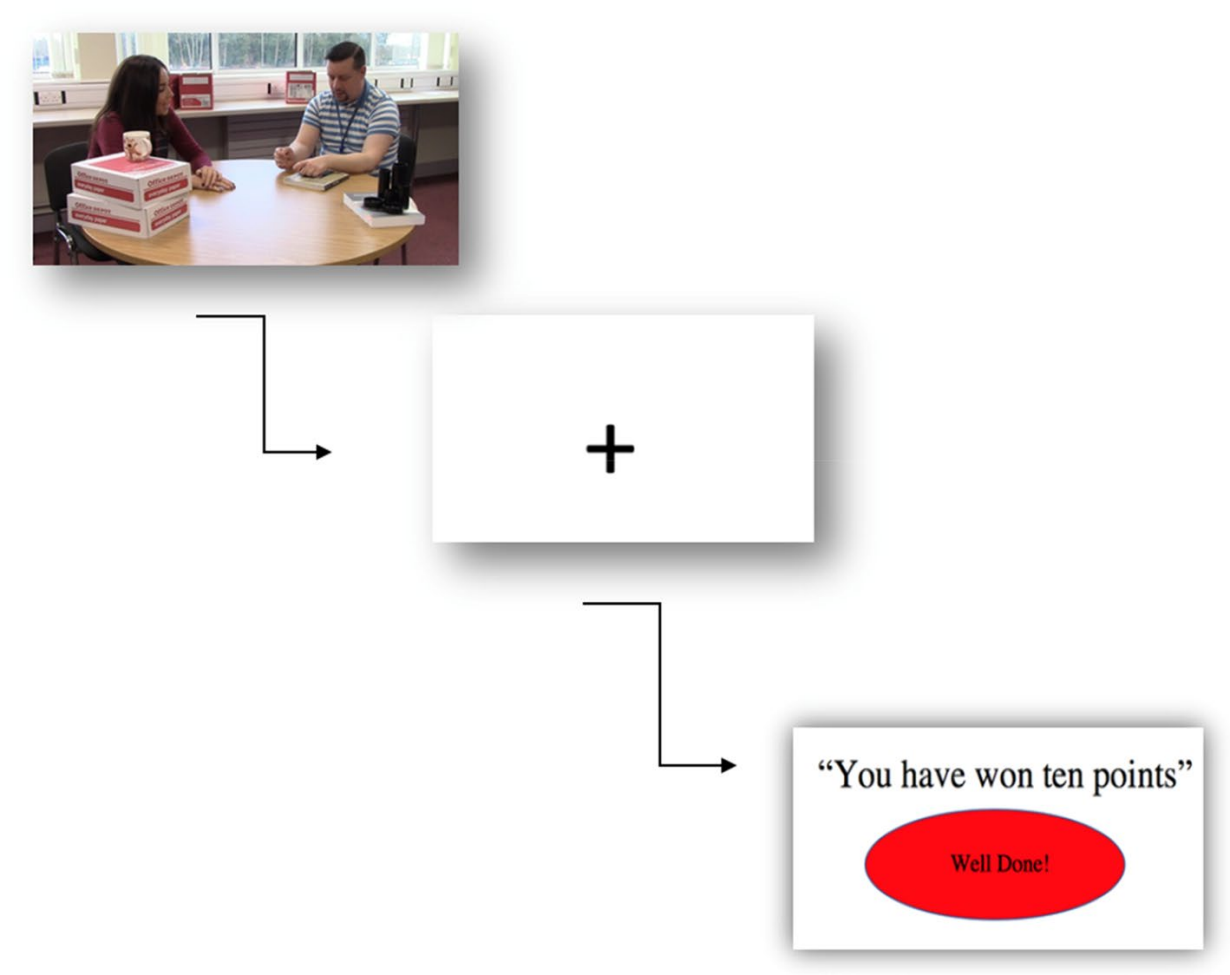
Depiction of training phase having met the face fixation contingency

On the day of testing, when a participant fixated on a face presented in the video clip for one second, this triggered a center cross to appear. When this center cross appeared on the screen, the researcher would then say to the participant ‘congratulations you have won ten points!’, immediately reinforcing this target behaviour (see Fig. 3). If the participant did not meet the criterion of looking at an image of a face for one second (during the 18–20 s running time of the clip), the center cross would appear, however the researcher would not comment and the experiment would progress onto the next video clip. Note that control participants did not complete this operant training phase but rather played an educational game on an iPad. Total testing time for this phase was no longer than three minutes. All participants scored the maximum amount of 100 points during this phase.

### Re-Test Phase

Finally, the re-test phase was a replication of the baseline phase with each participant wearing the SMI eye-tracking glasses when engaging in a dyadic and triadic interaction with the researcher/research assistant. Similar to the baseline phase, each live interaction was timed to last two and a half minutes. The ordering of interactions was randomized so that if a participant began with a dyadic interaction in the baseline, he/she would begin with a triadic interaction in the re-test phase and vice versa. This was to avoid sequencing effects with each participants’ gaze behaviour. Total testing time for the three experimental phases was twenty minutes.

## Results

### Data Reduction

SMI eye-tracking software known as BeGaze (SMI BeGaze™) recorded all monocular and binocular eye-tracking data for each participant so that it could be computed and anlaysed. When analysing the eye-tracking data, an Area of Interest (AOI) based approach was used. AOI’s define regions on the stimulus to quantify whether or how often each participant looked at particular regions (Holmqvist and Nystrom 2015). The AOI in the present study was defined as any face stimulus present during the live interactions conducted in a learning support classroom. Therefore, during the recording of a dyadic interaction (researcher and participant talking) the face AOI was defined as the researchers’ face; during a triadic interaction (researcher, research assistant, and child conversing), the face AOI’s were defined as the researchers’ face *and* the research assistants’ face. This approach made it possible to quantify the dimensions of gaze behaviour to be measured (duration, latency, and frequency) within an AOI (face stimulus) during both live interactions.

### Analytic Approach

All eye-tracking terminology and definitions used here are those stated by Holmqvist and Nystrom (2015). Given the current study followed a single- subject design, a visual analysis of multiple characteristics of the data was completed. However, we wished to align this study to recent advances in single- subject methods which recommends the inclusion of complimentary statistical analyses that answer the same research questions as that of the visual analysis (Kazdin 2019; What Works Clearinghouse 2020). Visual analyses of duration, frequency, and latency eye-tracking data were therefore supplemented with relevant statistical tests including effect size estimates corrected for small sample bias.

#### Duration: Visual and Statistical Analysis

**Fig. 4:**
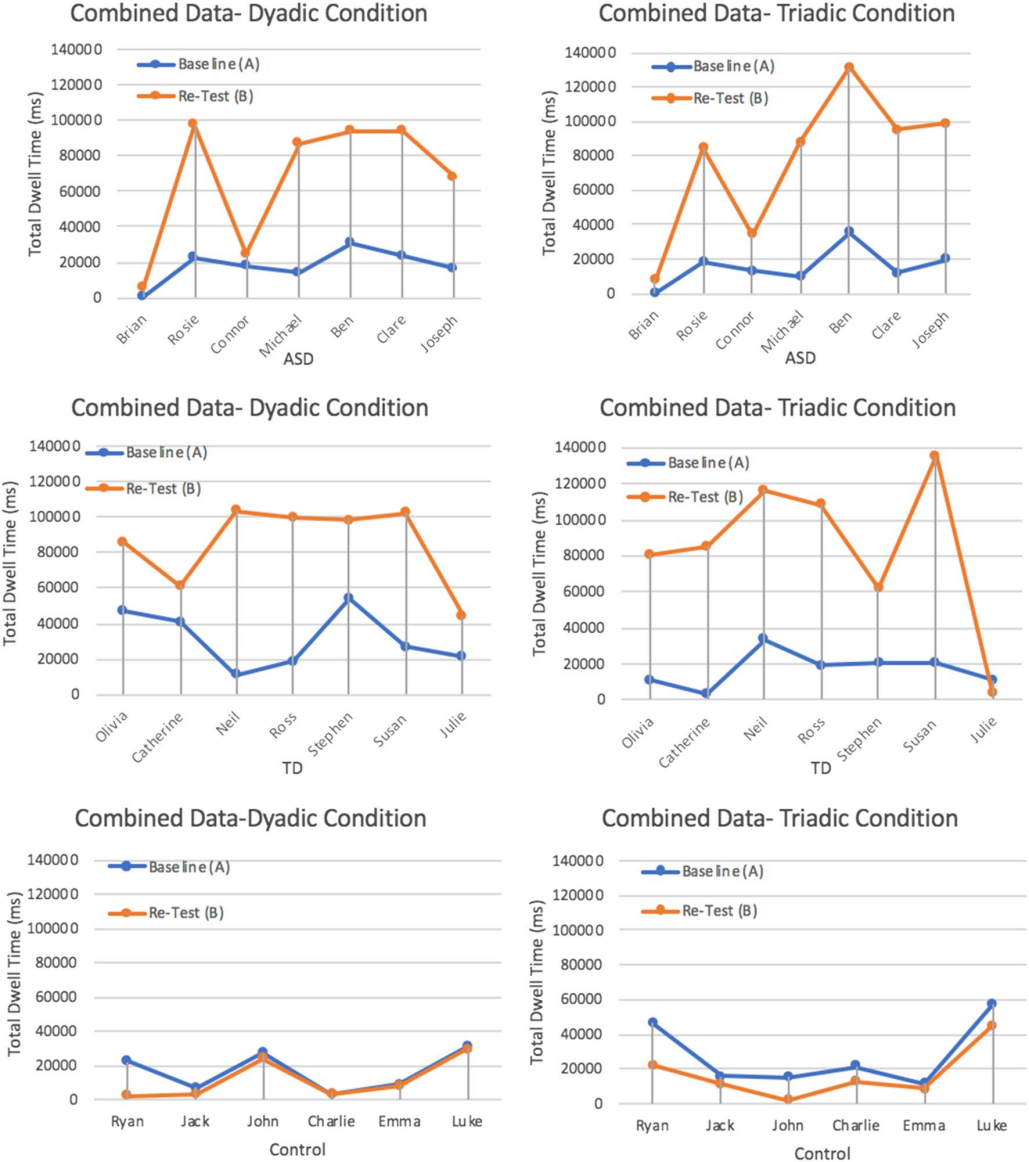
Total dwell time on face AOI during baseline and re-test phases for each participant

Duration of gaze behaviour was measured using the eye-tracking metric *total dwell time (ms)*. Total dwell time is a relative measure to calculate the amount of time spent looking at an AOI. Total dwell time was measured during the baseline (A) and re-test phases (B) to determine if there was a difference in the amount of time participants spent looking at faces (AOI’s) having completed the interim training phase. Results presented in Fig. 4 show individual eye-tracking data for all of the participants (ASD, TD, Control) across the two experimental phases.

Data in Fig. 4 were visually analysed to check for increases in level and trend between the experimental A-B phases. Each of the ASD participants spent longer looking at the face AOI in the re-test phase in response to operant training. This trend of increased dwell time on face AOI’s also extends to the TD participants in the dyadic condition and the triadic condition with the exception of Julie who looked less at face AOI’s in the re-test phase after completing operant training. A visual analysis of eye-tracking results for the control participants who did not complete any interim operant training phase showed a decline in looking time between baseline and re-test phases or similar looking behaviours as seen for Charlie, Emma and Luke.

A paired samples *t* test was conducted to determine if the visual difference between total dwell time (ms) scores for baseline and re-test phases were statistically significant for the dyadic and triadic interaction type. There was a significant difference in mean dwell time (ms) between baseline and re-test phases for ASD participants during the dyadic condition; *t*(6) = 4.23, *p* = 0.006. Overall, ASD participants looked at the face AOI for longer in the re-test phase (*M* = 66,959.83, *SD* = 37,618.06) than the baseline phase (*M* = 17,670.50*, SD* = 9606.36). This represented as a large effect size calculated using Cohen’s *d* = *1.80.* This significant difference was also observed in the triadic condition; *t*(6) = 4.87, *p* = 0.003. Overall, ASD participants looked at the face AOI’s in the triadic condition for significantly longer in the re-test phase (*M* = 77,186.66, *SD* = 41,954.41) than the baseline phase (*M* = 15,498.91, *SD* = 10,865.12). This represented as a large effect size, Cohen’s *d* = 2.01.

Paired samples *t* tests revealed this difference in mean dwell time (ms) was also significant for TD participants across both conditions. There was a statistically significant difference between mean dwell time between baseline and re-test phases for TD participants during the dyadic condition; *t*(6) = 4.86, *p* = 0.003. Overall, TD participants looked at the face AOI for longer in the re-test phase (*M* = 85,126.33, *SD* = 23,409.17) than the baseline phase (*M* = 31,562.14*, SD* = 15,840.97). This represented as a large effect size Cohen’s *d* = 2.68. This significant difference was also observed in the triadic condition; *t*(6) = 4.50, *p* = 0.004. Overall, TD participants looked at face AOI’s in the triadic condition for significantly longer in the re-test phase (*M* = 84,752.96, *SD* = 43,183.99) than the baseline phase (*M* = 17,160.66, *SD* = 9681.06) in response to operant training. This represented as a large effect size, Cohen’s *d* = 2.16.

For control participants who did not complete operant training, no significant difference was found for these participants between baseline and re-test phases for either dyadic or triadic conditions; *t*(5) = 1.48, *p* = 0.19 and *t*(5) = 3.57, *p* = 0.16 respectively. Overall, paired samples *t* tests confirm the visual analysis of duration data; all ASD and TD participants looked for significantly longer in the re-test phase in response to operant training, with no significant difference observed for control participants.

#### Latency: Visual and Statistical Analysis

**Fig. 5:**
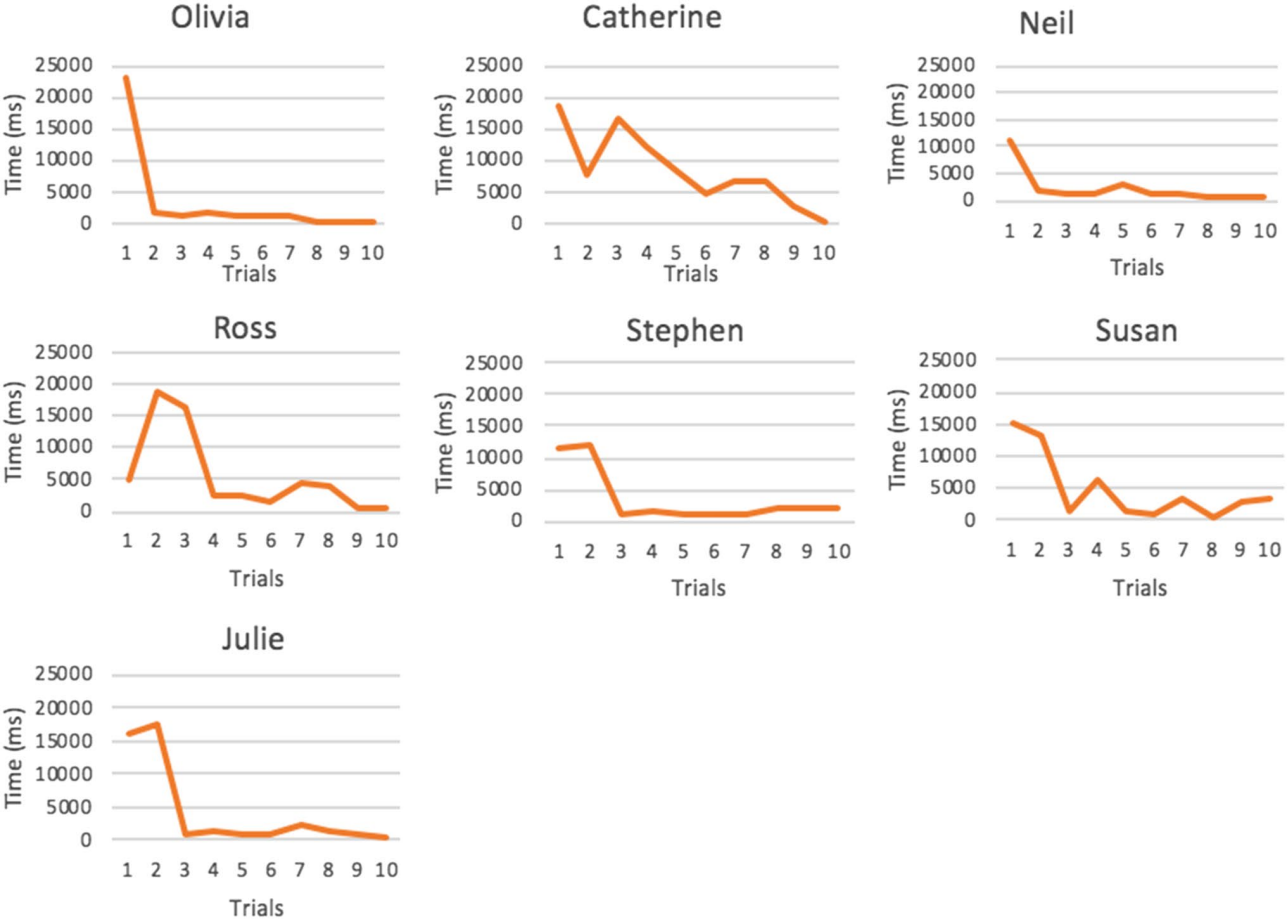
Latency data during training phase for TD participants

Latency of gaze behaviour was defined as the amount of time (ms) taken from the trial starting until the participant triggered the center cross screen-which signaled to the researcher to positively reinforce this target behaviour. Recall that triggering this center cross screen relied on the participant meeting the criterion of fixating on an image of a face present in the dynamic video clip for 1 s. Figure 5 shows latency data visually presented for each ASD participant.

A visual analysis of latency eye-tracking data provides an insight into behaviour change over time in response to the reinforcement contingency during the training phase. Latency data for each TD participant who completed training are shown in Fig. 5. There is a general downward sloped pattern as trials progress from 1 to 10. Therefore, each participant is responding to looking at the face stimulus in the training phase with reduced latency in response to reinforcement. This downward trend is particularly strong for Olivia, Neil, Stephen and Julie with more variability found in the data series for Catherine, Ross and Susan. For all TD participants the longest latency can be found in the initial trials when participants are making associations between the antecedent, behaviour and consequence.

**Fig. 6:**
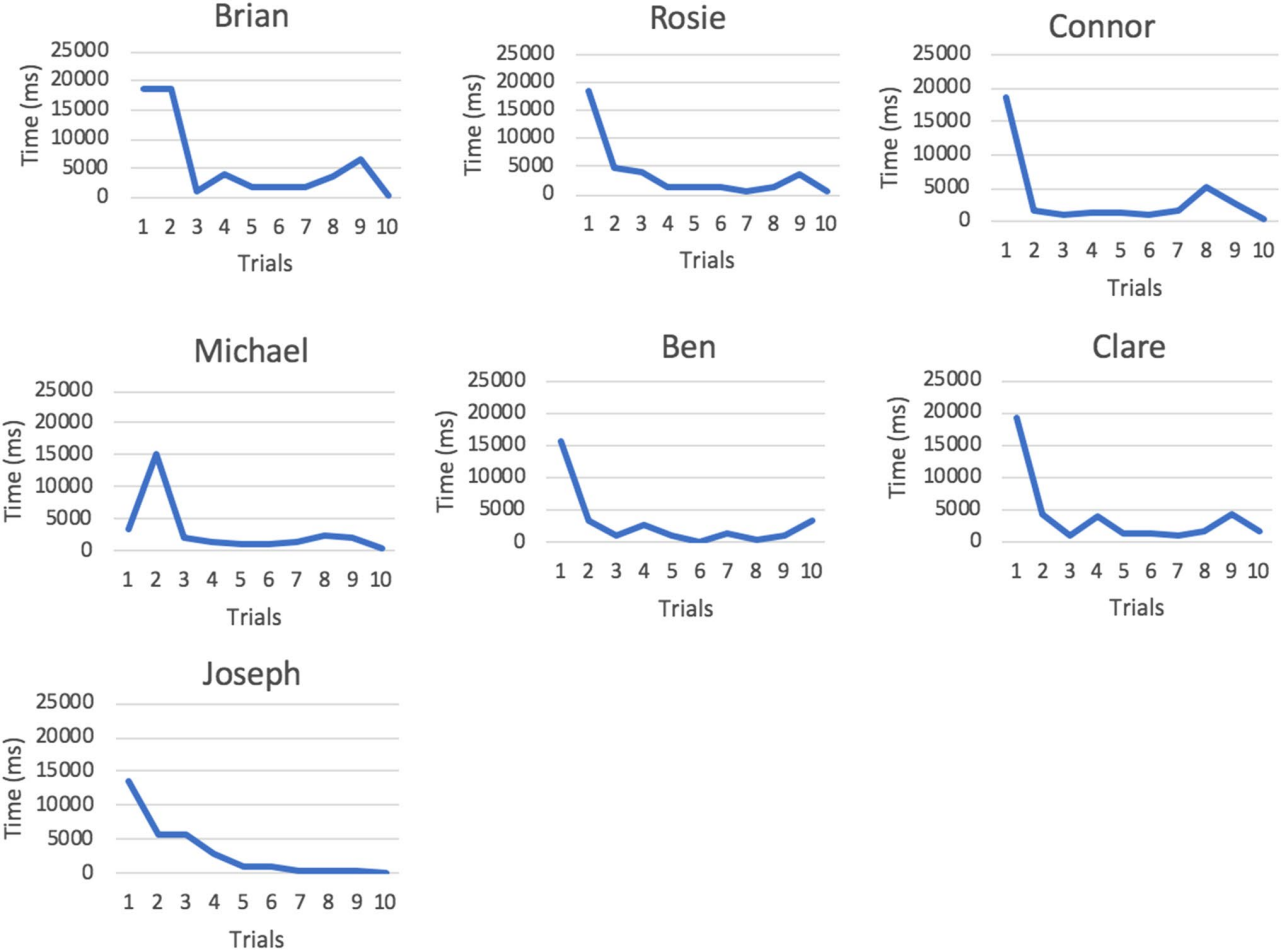
Latency data during training phase for ASD participants

A visual analysis of latency eye-tracking data for ASD participants who completed training shows a similar trend of behaviour change in response to the delivery of reinforcement as TD participants. The data of each ASD participant in Fig. 6 shows a downward sloped trend, highlighting the reduced latency of fixating on a face stimulus as reinforcement is delivered from trials 1 to 10. For Michael and Joseph this is a distinctively sharp behaviour change.

For latency data, measured at the ordinal level, the Wilcoxon-Rank test was used to determine if there was a significant difference in latency behaviour between trial 1 and trial 10 during the training phase. For ASD participants (*n* = 7) there was a statistically significant difference between trial 1 and trial 10 latency scores Z = 2.37, *p* = 0.018. The median latency score at trial one was 15,551 ms and by trial 10 was 378 ms (interquartile range 15,851–325 ms). Similarly, for TD participants, there was a statistically significant difference between trial 1 and trial 10 latency scores Z = 2.67, *p* = 0.015. The median latency at trial one was 14,998 ms and by trial 10 was 401 ms (interquartile range 18,094–3181 ms). Both statistical tests corroborated the visual behaviour change (seen in Figs. 5 and 6) of reduced latency to fixate on face AOI’s in response to operant training.

#### Frequency: Visual and Statistical Analysis

**Fig. 7:**
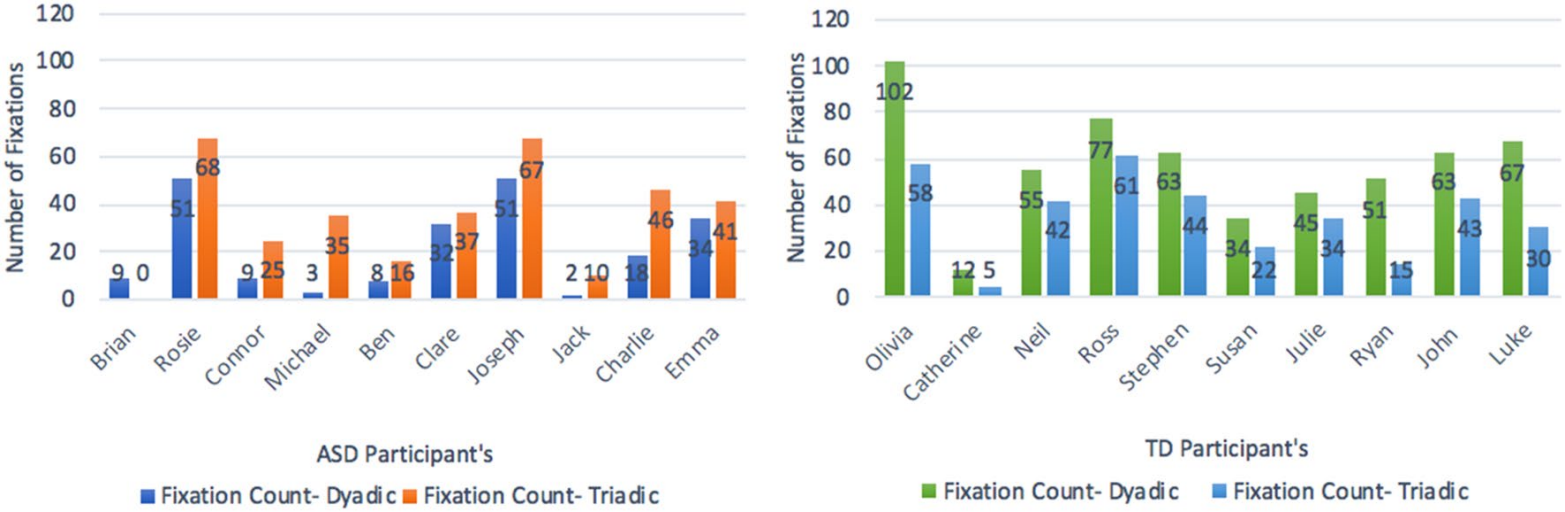
Frequency data during baseline dyadic/triadic conditions for each participant

Baseline frequency data for dyadic and triadic interaction types are presented in Fig. 7. A visual analysis of this data showed variability within each group of participants. Nine out of ten ASD participants (with the exception of Brian) made a higher number of fixations in the triadic condition than the dyadic condition. This difference was particularly evident for Rosie, Conor, Michael and Charlie. Visual analysis of the ten TD participants displayed a trend opposite to ASD participants. Each of the TD participants made a higher number of fixations in the dyadic condition than the triadic condition. This was particularly evident for Olivia, Ryan and Luke. Catherine’s fixation data showed a lower fixation count across both conditions compared to the other TD participants.

Kolmogorov–Smirov Tests for normality confirmed baseline fixation count data followed a normal distribution and so a repeated measures ANOVA (RM-ANOVA) was ran for each set of participants (ASD and TD). There was a significant main effect of interaction type (dyadic vs. triadic) on fixation count for ASD participants: *F*(1,9) = 11.16, *p* = *0.0*09*.* The number of fixations were significantly higher in the triadic condition (*M* = 34.50, *SD* = 22.56) than the dyadic condition (*M* = 21.90, *SD* = 19.12) for ASD participants. This represented as a moderate effect size, Cohen’s *d* = 0.61.This main effect of interaction type was also found for TD participants: *F*(1,9) = 28.14, *p* < *0.001.* Conversely, the number of fixations were significantly higher during the dyadic condition *(M* = 56.90, *SD* = 24.35) than the triadic condition (*M* = 35.40, *SD* = 17.93) for TD participants. This represented as a large effect size of Cohen’s *d* = 1.2. The opposing directions of this main effect for both ASD and TD participants compliments findings from the initial visual analysis of single- subject data presented in Fig. 7.

#### Social Functioning

Skewness statistics were assessed for SRS *T*-Scores and baseline total dwell time (ms) for ASD participants and showed neither differed significantly from a symmetrical distribution. A Pearson’s correlation found there was a strong, negative, statistically significant correlation between SRS *T*-Scores and baseline total dwell time (during both interaction types) on face AOI’s; *r*(10) = − 0.73, *p* = *0.0*24*.* Higher SRS-2 scores indicating a higher degree of social difficulties were related to a reduced amount of time spent looking at face AOI’s during ‘real-world’ social interactions for ASD participants.

## Discussion

The current study investigated gaze behaviour of children with ASD and TD children during live social interactions in a ‘real-world’ classroom setting. This study aimed to extend previous lab-based research (Falck-Ytter 2015; Noris et al. 2012; Noris et al. 2011; Thorup et al. 2016; Vabalas and Freeth 2016) by conducting eye-tracking research in an ecologically valid classroom setting. The current study also aimed to investigate if gaze behaviour could be changed in a ‘real-world’ setting using operant training. Again, this was a novel addition to previous eye-tracking research which, thus far, has failed to engage with developing gaze behaviour interventions that help teach typical gaze behaviour to children with ASD and improve visual attention during social interactions. A single-subject methodological design was used to capture the individual gaze behaviour patterns of children, an important consideration given the heterogeneity that exists in ASD populations. In keeping with recent developments in single-subject designs, relevant statistical analyses were completed to compliment the visual data analysis approach used in single-subject designs (Kazdin 2019).

### Response to Research Question 1

The first research question outlined in the current study asked; *does an atypical pattern of gaze behaviour, previously found amongst individuals with ASD during live interactions, occur in a ‘real-world’ classroom setting?* This research question was answered using eye-tracking data from the baseline phase only to provide a raw and representative measure of gaze behaviour prior to any operant training. A visual analysis of fixation count data, a measure of gaze behaviour frequency, showed that all ASD participants made a higher number of fixations on face stimuli presented during triadic interactions, compared to a single face stimulus during a dyadic interaction (see Fig. 7). This visual analysis was supported by a RM-ANOVA confirming a significant main effect of interaction type (dyadic or triadic) on the number of fixations made on AOI’s for ASD participants. This pattern of results proved atypical when considered alongside eye-tracking data for TD participants; all TD participants fixated more often on a face AOI during a dyadic interaction compared to a triadic interaction. Statistical analyses revealed a significant effect of interaction type on fixation count, in an opposing direction to ASD participants—significantly more fixations were made on AOI’s during a dyadic interaction compared to a triadic interaction.

This atypical finding for ASD participants when engaging in live dyadic or triadic interactions, should be considered in relation to the *communicative intent* each interaction depicts. For example, dyadic interactions depicted in the current study represented high communicative intent—the researcher was *directly* engaging with the child participant, whereas triadic interactions represented low communicative intent—the researcher and research assistant were steering conversation, as the child participant looked on and occasionally had an input. The finding of reduced fixations on a face stimulus for ASD participants during an interaction of high communicative intent supports the notion that the potential for social interaction alone can lead people to avoid looking at others (Hessels 2020). It also supports previous eye-tracking research which has found that the nature of a social situation (being interactive or not) affects the distribution of gaze behaviour across a social scene between ASD and TD individuals (Hanley et al. 2017). Previous studies have related this to interactive social interactions demanding immediate understanding of social cues and emotional expressions (Hanley et al. 2017) and difficulties individuals with ASD have with integrating verbal and visual inputs of information (Anderson et al. 2006). The fact these gaze behaviour patterns have now been uncovered during interactions in a classroom, raises important considerations for how children with ASD might best respond to different pedagogic teaching styles; interacting directly with one other person face to face may affect his/her gaze behaviour and therefore understanding of the social situation.

This disparity between the number of fixations children with ASD made during a dyadic interaction compared to a triadic interaction might concern *joint attention*. As previously outlined in this study, children with ASD have impairments in joint attention; described as two persons actively sharing attention to an object or throughout a conversation (Adamson and Bakeman 1984; Scaife and Bruner 1975). In many ways, these gaze behaviour findings may offer some explanation as to why children with ASD present with joint attention issues (Mundy and Crowson 1997; Mundy et al. 1996). Simply put, if a child is not looking at a face, they cannot engage in joint attention, and vice versa. Therefore, it would be worthwhile for joint attention studies to consider some of the variables presented here which are increasingly shown to affect the gaze behaviour of children with ASD (number of actors, level of communicative intent etc.). By considering the gaze behaviour atypicalities and profiles amongst children with ASD, a deeper understanding into *why* these children fail to engage in joint attention might be uncovered. We would equally suggest that a welcomed addition to live eye-tracking research might be a measure of joint attention skills (using the Early Social Communication Scales, ESCS, Mundy et al. 2003) for children with ASD to be interpreted alongside eye-tracking data.

Research question 1 was explored further by including a measure of the degree of social functioning (SRS-2, Constantino and Gruber 2005) for ASD participants. The correlation analysis represented as a strong, statistically significant, negative relationship between SRS severity and time spent fixating on AOI’s during the baseline phase. In other words, as autism severity increased, time spent fixating on face AOI’s decreased for ASD participants. This trend supports previous studies which have shown a link between ASD symptoms and social attention towards social stimuli (Bird et al. 2011; Chawarska et al. 2012; Klin et al. 2002). It also supports the efficacy of measuring symptom severity amongst ASD populations given the growing body of research that has found links between differential gaze patterns for ASD children towards different facial expressions depending on autism severity (Matsuda et al. 2015). The internal consistency demonstrated here between a teacher report of a child’s behaviour (based on classroom observations over past 6 months) and eye-tracking data gathered in a ‘real-world’ setting of a classroom, confirms how eye-tracking data can complement and act as an objective measure to better understand the social behaviour of a child with ASD. It highlights the fruitful union of psychometric tests and ‘real-world’ eye-tracking data in building a more comprehensive behavioural profile for a child with ASD.

### Response to Research Question 2

The second research question asked; *can gaze behaviour be improved in this ‘real-world’ setting using an operant training tool?* The total dwell time eye-tracking metric helped address this question to detect any changes in the *duration* of a participants’ gaze behaviour from baseline to re-test phases (having completed a training phase). The visual analysis from the total dwell time (during dyadic and triadic interactions graphs (Fig. 4) showed that all ASD and TD participants (with the exception of Julie) gaze behaviour changed so that they look for longer at the researchers’ face (during a dyadic interaction) and at the faces of the researcher and researcher assistant (during a triadic interaction) in the re-test phase in response to training. These results are particularly interesting given numerous ASD participants (Michael, Ben, Clare, Joseph, Rosie) spent up to four times longer looking at the researchers’ face during the re-test dyadic interaction, with similar differences during the re-test triadic interaction. Note that live eye-tracking video from the triadic condition showed Julie was highly distracted by pencils and pens sitting on the desk in the classroom; most likely accounting for no behaviour change in the re-test phase.

Paired samples *t* tests confirmed this visual difference in dwell time duration between dyadic and triadic conditions for ASD participants was statistically significant in response to operant training. These differences represented as large effect sizes for both dyadic and triadic conditions (*d* = 1.80; *d* = 2.01 respectively). From this it is reasonable to suggest that gaze behaviour is an operant capable of changing in response to reinforcing contingencies, supporting prior research by Steingrimsdottir and Arntzen (2016). To be sure any gaze behaviour change was indeed attributable to the operant training and to rule out any confounding explanations (for example, familiarity), control participants in this pilot study offer important confirmation. The visual analyses of dwell time data coupled with the statistical *t* tests analyses showed no significant differences in time spent looking at faces between the baseline and re-test phases for control participants. Therefore, we can conclude with greater certainty that the increase in dwell time on face stimuli (AOI’s) for participants who completed the training phase was in fact due to the operant training delivered on the eye-tracking equipment. It also confirms the functional role that reinforcement played in this study; participants were able to generalize this operant training delivered on desktop eye-tracking equipment into the ‘real-world’ interactions during the re-test phase.

Latency data collated during the training phase also provided a response to research question 2. A visual analysis of the slope in these training graphs (Figs. 5, 6) highlighted that *all* ASD and *all* TD participants began to respond to looking at a face with reduced latency. In other words, the operant approach of reinforcing a target behaviour of looking at a face for 1 s, increased the likelihood of this behaviour occurring again (with reduced latency) in the subsequent trials. Non-parametric Wilcoxon-rank tests confirmed these downward sloped trends represented a significant difference over time between trial 1 and trial 10 in the direction of lower/reduced times to trigger the reinforcement. Considering duration and latency eye-tracking analyses together, this study reaffirms findings from Isaksen and Holth (2009). Similar to that study, we have successfully paired rewards with a target behaviour to arrange a contingency that increased the likelihood of that behaviour occurring again. This links nicely to the recent prospect of accelerating the pace of ASD research and treatment with the use of innovative technology (Chita-Tegmark 2016; Goodwin 2008) and opens many avenues for researchers to develop, further, technology based training tools to benefit the social skills of children with ASD.

## Limitations

The main limitation of the current study is the small sample size. Whilst this was a single- subject research design and statistical analyses were adjusted for small sample size, building an evidence base for this operant training tool can only be achieved with more replicability. The basic single- subject design used here (A–B phases with an interim training phase) might also be extended to include more robust single- subject designs (ABAB; multiple baseline; changing criterion designs) with multiple training phases as opposed to the limitations of one in the current pilot study.

## Future Directions

The current study has begun to open the possibility of improving gaze behaviour of children with ASD using the very eye-tracking technology that researchers have used for decades to analyse it. Follow up questions that were not within the scope of this current study might include; can gaze behaviour change be maintained over time? and can gaze behaviour change be generalised to other ‘real-world’ settings, for example, the home environment? One further interesting area for future research might be to use older participants, for example, adolescents and adults, as an extension of the child-based sample of the current study. Using older participants who have perhaps developed hard-wired compensatory strategies around gaze behaviour over the years (Livingston et al. 2019) may be more resistant to gaze-behaviour training. Finally, the inclusion of a qualitative component to gage if participants’ understanding of the social interaction improves as a result of allocating their gaze to faces more often in the re-test phase, would be a welcomed addition in future research.

## Conclusion

In conclusion, the current study has advanced previous eye-tracking research beyond the confines of conducting live interactions in controlled lab environments. By conducting research in an ecologically valid setting, a school classroom, findings from the eye-tracking data can be interpreted through a representative lens. The application of ABA principles to understand and indeed change gaze behaviour has been piloted in this study and shows promising preliminary results for future larger scale and longitudinal research to be conducted. In addition to this, the single-subject nature of this current study helps researchers and clinicians to appreciate the unique gaze behaviour profile of children with ASD, something that is overlooked in large between- group statistical analyses common in eye-tracking research. It is hoped the current study will help pave the way for intervention research to improve the gaze behaviour of children with ASD and the quality of their daily social interactions.
